# The Power of the Pedigree: Cascade Screening in Familial Hypercholesterolemia

**DOI:** 10.1016/j.jacadv.2024.101201

**Published:** 2024-08-13

**Authors:** Jared A. Spitz, Rebecca Miller, Jaideep Patel

**Affiliations:** a Inova Schar Heart and Vascular, Fairfax, Virginia, USA; b Inova Genetics Department, Fairfax, Virginia, USA; c Johns Hopkins Hospital, Baltimore, Maryland, USA; d Ciccarone Center for the Prevention of Cardiovascular Disease, Johns Hopkins University School of Medicine, Baltimore, Maryland, USA

**Keywords:** familial hypercholesterolemia, cascade testing, implementation strategies, family communication

**Figure:**
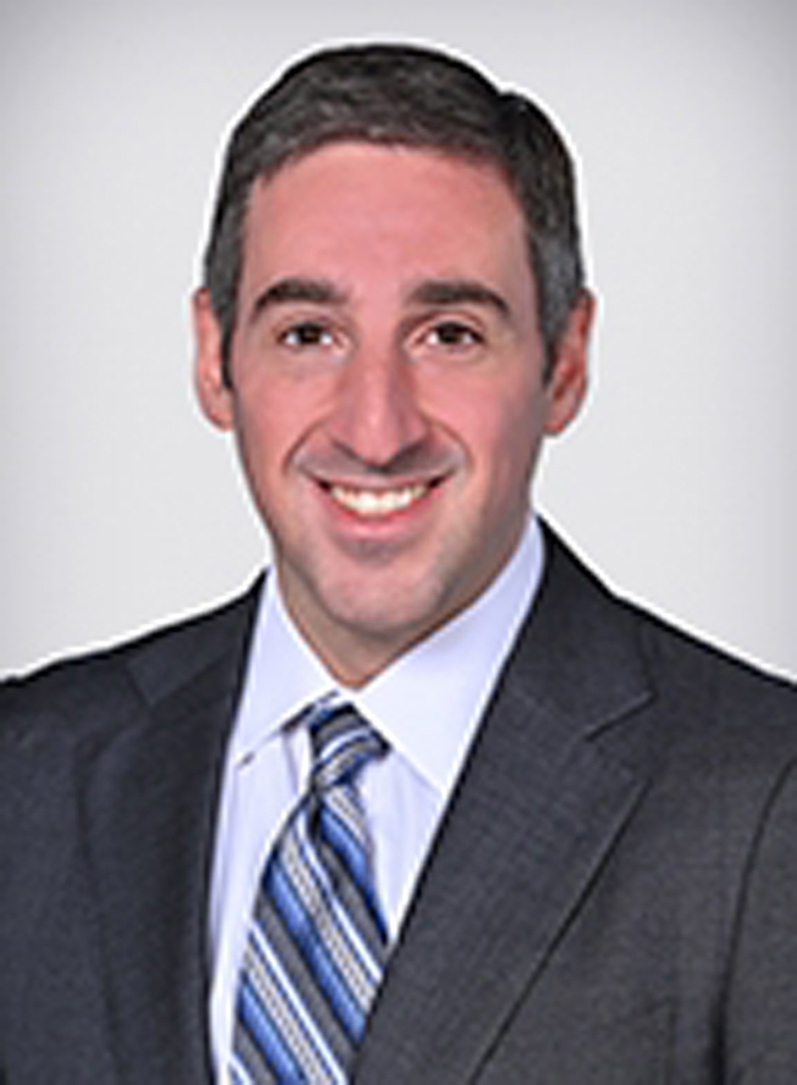

Familial hypercholesterolemia (FH) is an autosomal dominant genetic disorder characterized by lifelong elevated levels of cholesterol.^1^, ^2^, ^3^ Despite widespread prevalence (∼1 in 250 people for the heterozygous form), it remains severely underdiagnosed, with estimates that <10% of affected individuals are aware of their diagnosis.^2^^,^^4^ Considering the linear relationship of elevated low-density lipoprotein cholesterol to atherosclerotic cardiovascular disease, FH patients experience accelerated atherosclerosis in the setting of cumulative lifetime exposure to high low-density lipoprotein cholesterol concentrations. FH can be diagnosed based on clinical criteria;^5^ however, if feasible, genetic testing for causative genes should be undertaken^1^^,^^3^ with testing for pathogenic variants in three primary genes: *LDLR*, *APOB*, and *PCSK9*.

Cascade screening allows for testing of family members of the index patient (the proband) who are at-risk based on their relationship to the patient; this can be done through a combination of lipid profile screening and genetic testing.^1^^,^^3^ Among those with genetically confirmed FH, cascade screening represents a simple, cost-effective method for identifying additional cases in a family. This ultimately leads to earlier diagnoses of FH and promotion of lipid-lowering therapy.^1^^,^^6^^,^^7^

As an example of its effectiveness, a cascade screening program in the Netherlands, where genetic results were shared with a national foundation who then contacted the proband to help facilitate cascade screening, helped identify 70% of cases nationwide.^4^ In the United States, it is estimated that only ∼9% of probands had at least one relative screened,^8^ which may stem from significant barriers to cascade screening:^2^^,^^6^^,^^9^ 1) underdiagnosis of the index case (knowledge of the disorder, awareness of current guidelines, challenges of deciphering lipid and family history, patient opposition to screening, ineffective communication of genetic information among family members); 2) the federal Health Insurance Portability and Accountability Act prohibits direct contact of at-risk relatives unless prior authorization from the proband has been given (ie, privacy laws); 3) providers and payers tied to poor reimbursement; and 4) concerns around genetic discrimination and stigmatization tied to the ability to obtain health insurance once a diagnosis is made.^2^^,^^4^^,^^10^, ^11^, ^12^

Given the significant gap between recommendations for cascade screening and the implementation of this screening, more information is needed to develop an evidence-based, best practice approach. One of the crucial barriers is the initiation of cascade screening after index patient diagnosis. In this issue of JACC: Advances, Jones et al. present an interesting, real-world, pragmatic study evaluating strategies for improvement in cascade testing in those with genetically confirmed FH.^13^ In this novel implementation study, proband patients with FH were offered three options for engaging potentially affected family members: a packet with pertinent information for a proband to send relatives, an electronic link sent to family members leading to a chatbot offering information about the diagnosis and cascade screening, or direct contact. In this study, utilization of a strategy by the proband led to a >6-fold increase in cascade testing compared to no strategy selected at all. Notably, the two most effective strategies were the chatbot and direct contact; however, these were also the least frequently chosen option.

The main strength of this study is its pragmatic design. A “know-do” gap exists in cascade screening for FH wherein we know that cholesterol testing and cascade screening are needed, but how to best implement this is not known and understudied.^8^ This study evaluates several potential options to help spur cascade screening. Notably, it compares a commonly employed strategy of the use of a letter of explanation to be given to probands’ relatives with one of the most effective measures-based on international programs-of direct contact of potentially affected relatives.^2^^,^^8^^,^^9^ Importantly, the study also investigates the potential use of a chatbot and helps evaluate how the use of telehealth and artificial intelligence will impact future care in this field.

However, a number of limitations are noted for this study.^13^ The study occurred within the Geisinger health care system and covers a largely rural population, involving a majority of White and non-Hispanic, English-speaking adults. These both limit the generalizability of these results to broader populations. This is an important gap, as minority populations in the United States are even more underdiagnosed with FH than White patients.^8^ Additionally, this study only assessed cascade screening in those who had confirmatory genetic testing and did not evaluate screening of at-risk relatives when the FH diagnosis was made by clinical criteria.

There are a number of key takeaways from this study.^13^ Selection of a cascade screening strategy led to significantly greater uptake of cascade testing compared to no strategy being selected. While this may intuitively make sense, this reinforces the need for education of proband patients about the hereditary nature of the condition, explanation about the role of cascade screening, and guidance of patients to share information with their relatives and encourage cascade screening. Also notable is that in patients who had consultation with a genetic counselor, subsequent selection of a cascade screening strategy was much higher. In other pragmatic studies, the presence of a genetic variant identified in a proband led to higher adoption of cascade screening.^14^ In this study, family member contact could be initiated via genetic counselors or a clinician.^14^ That the occurrence of a genetic counseling appointment led to higher rates of follow-up cascade screening strongly suggests the integral role genetic counselors have in communicating the diagnosis, the hereditary implications, and in facilitating cascade screening.

This study by Jones et al. adds to the growing list of pragmatic studies about how to better achieve the goal of cascade screening after a diagnosis of FH.^13^ There are several implications that should help shape future policy discussions around genetic testing. The first is that genetic counselors play a crucial role in the diagnosis and follow-up of genetic diseases. Policies within institutions and at a state or federal level should be established that help enshrine the scope of practice of genetic counselors as co-equal members of a health care team; the corollary of this should be changes in insurance coverage and reimbursement that support this. Second, direct contact with relatives proved to be the most effective tool for ensuring cascade screening. Given concerns around patient safety and discrimination based on having genetic conditions, a policy framework addressing those concerns is imperative. Banning discrimination for life and disability insurance would be a good first step. Tools for increasing proband consent for direct contact for cascade screening should be further studied within the United States; many strategies have been used outside of the United States, but these will need to be tailored to comport with the U.S. privacy concerns and regulations. Third, the use of telehealth and chatbots raises the specter of increasing access to specialty providers by those patients in more rural or health care-limited settings. However, this requires telephone and internet access, and policies aimed at facilitating this access for health care-related purposes need to be addressed.

This study takes the field further in our understanding of the implementation of cascade screening for FH. It is imperative that future research continue focusing on the role of genetic counseling and integration of genetic counselors into multidisciplinary teams. Understanding barriers to sharing genetic information at a patient level needs to be understood so that solutions can be implemented to address them. Even so, there remains a staggeringly high rate of underdiagnosis of FH, and research into the lack of education and how to overcome this remains paramount.

## Funding support and author disclosures

The authors have reported that they have no relationships relevant to the contents of this paper to disclose.
